# High miR-449b expression in prostate cancer is associated with biochemical recurrence after radical prostatectomy

**DOI:** 10.1186/1471-2407-14-859

**Published:** 2014-11-21

**Authors:** Martin Mørck Mortensen, Søren Høyer, Torben Falck Ørntoft, Karina Dalsgaard Sørensen, Lars Dyrskjøt, Michael Borre

**Affiliations:** Department of Molecular Medicine, Aarhus University Hospital, Brendstrupgaardsvej 100, 8200 Aarhus N, Denmark; Department of Urology, Aarhus University Hospital, Brendstrupgaardsvej 100, 8200 Aarhus N, Denmark; Department of Pathology, Aarhus University Hospital, Nørrebrogade 44, 8000 Aarhus C, Denmark

**Keywords:** Prostate cancer, microRNA, miR-449, Biomarker, Biochemical recurrence, Prostatectomy

## Abstract

**Background:**

Prostate cancer is one of the leading causes of cancer death amongst men in economically advanced countries. The disease is characterized by a greatly varying clinical course, where some patients harbor non- or slowly-progressive disease, others highly aggressive disease. There is a great lack of markers to differentiate between aggressive and indolent disease. Markers that could help to identify patients needing curative treatment while sparing those who do not.

**Methods:**

MicroRNA profiling of 672 microRNAs using multiplex RT-qPCR was performed using 36 prostate cancer samples to evaluate the association of microRNAs and biochemical recurrence after radical prostatectomy.

**Results:**

Among 31 microRNAs associated with recurrence, we identified miR-449b, which was further validated in an independent cohort of 163 radical prostatectomy patients. Patients expressing miR-449b had a significantly higher risk of recurrence (HR = 1.57; p = 0.028), and miR-449b was shown to be an independent predictor of recurrence after prostatectomy (HR = 1.9; p = 0.003) when modeled with known risk factors of recurrent disease in multivariate analysis.

**Conclusion:**

High miR-449b expression was shown to be an independent predictor of biochemical recurrence after radical prostatectomy.

## Background

There is a great unmet need for better diagnostic as well as predictive tools in prostate cancer (PC) needed for discerning aggressive PC from indolent PC. Although this disease has the second highest cancer mortality amongst men, approximately 250,000 worldwide [1]. The prevalence hereof, as indicated by autopsy studies, is even higher and reported as being up to 12-54% of all men aged over 50 years [2]. Early diagnosis is imperative since curative treatment is only possible in non-metastatic PC. This has led to the widespread use of screening for PC using Prostate Specific antigen (PSA) testing, but since the prevalence of non-lethal, non-progressive PC is very high, screening leads to a substantial risk of overtreatment [3]. Biomarkers associated with aggressive PC have the potential of improving existing prediction models for identifying patients with aggressive disease and thus aid patients and physicians in deciding between curative treatment and active surveillance [4]. Furthermore, biomarkers associated with aggressive disease could be used for monitoring patients in active surveillance and be used as a trigger for offering curative treatment if the biomarker level changes [5].

In brief, microRNAs are small 18 to 25 nucleotide RNA molecules that function as regulators of gene expression through incorporation into the RNA-induced silencing complex (RISC). The incorporated microRNA binds to mRNA sequences with complementary to the microRNA. Following binding, the Argonaute (Ago) protein also incorporated in RISC cleaves the mRNA strand and thus causes degradation of the target transcript and thereby silences the gene [6]. Through this post transcriptional process miRNA can influence gene expression and thus regulate various biological processes. Since the key biological differences between cancer cells and their normal counterparts are the initiation and utilization of cellular processes like increased proliferation, immortalization and invasive properties, altering gene expression is central in tumorigenesis [7]. Previous studies have shown that miRNA can be central in orchestrating the altered gene expression necessary for the cell to undergo transformation to a cancer cell [6]. As each miRNA can have many different putative target mRNAs, a miRNA can essentially be the central tumorigenic factor [8] or it can function through inhibiting mRNAs that in turn function as either oncogenes or tumor suppressors [9, 10].

miRNAs are generally more stable and easier to measure in biological material where degradation is an issue compared to mRNA [11, 12]. Therefore, miRNAs may prove to be good biomarkers for diagnosis as well as for monitoring PC. It has been shown in several studies that miRNA can be assayed in blood from PC patients and that diagnostic miRNAs can be identified [13, 14].

Several studies have shown that miRNAs are aberrantly expressed in PC and several specific miRNAs have been implicated in PC development [15–19]. While most studies of miRNA in PC have focused on miRNAs differentially expressed between normal and cancerous prostate tissue, only a few studies have focused on miRNA expression associated with aggressive disease. A number of different surrogate end-points for aggressive PC have been used in the studies like presence of perineural invasion [20], Gleason grade [20, 21], extra prostatic growth [16] and risk of recurrence [22]. These differences in end-points, and the limited power of some studies due to few analyzed samples, are probably the main reasons that reproducing results is difficult, as described by Coppola et al. [23].

Here we performed microRNA profiling using multiplex qPCR on laser micro dissected material from 36 PC patients to identify microRNAs associated with recurrence after radical prostatectomy (RP). We validated the association with recurrence of the top ranked miR-449b in an independent cohort of patients using singleplex RT-qPCR.

## Methods

### Ethics statement

The study was approved by the Central Denmark Region Committees on Biomedical Research Ethics case number 2002-41-2640. Informed written consent was obtained from all patients.

### Clinical samples

Samples for this study were provided by the Aarhus prostate cancer project consisting of all patients undergoing radical prostatectomy at the Dept. of Urology Aarhus University Hospital from 1995 to present day. Samples included in the study were from 2003 to 2007. Clinical data were collected prospectively and recurrence status for all patients in the study was updated prior to inclusion in the study. The prostatectomy specimens were examined by an experienced uro-genito-pathologist assessing pathological stage (pT) and tumor differentiation scored according to Gleason. No re-review of the Gleason grade was performed. Serum PSA was measured prior to surgery by automated immunoassay using DPC Total PSA Immulite and expressed in ng/mL. Clinical follow up after surgery was conducted by PSA measurements at 3, 6, and 12 months postoperatively and thereafter biannually. Subsequent biochemical failure was defined as two consecutive measurements of PSA > 0.2 ng/mL. Needle biopsies were taken from the surgical prostatectomy specimen and immediately snap frozen.

For the validation phase, samples cores 1.5 mm in diameter were taken from formalin fixed paraffin embedded (FFPE) radical prostatectomy specimens. The patient cohort [24, 25] and sampling protocol for RNA extraction have previously been described. All clinical information from the patient cohort was updated regarding recurrence status prior to inclusion in the current study. Patients with at least 4 years recurrence free survival and patients with proven biochemical recurrence were included.

### Laser micro dissection and RNA extraction

Survey slides of the biopsies were examined and the carcinoma cells identified. Since all material was reevaluated by an experienced uro-genito-pathologist to ensure that only tumor tissue was included. Subsequently, slides were stained with cresyl-violet 1% and the carcinoma cells were laser micro dissected using the PALM laser microbeam system. RNA extraction was performed using RNeasy® Micro kit from Qiagen (Germany). Flow through from the RNA extraction contains RNA fragments that are shorter than 120 nucleotides in length, thus the total microRNA fraction was contained within the flow through. A second microRNA extraction was performed on the flow through with RNeasy micro kit optimized for extracting micro RNAs.

RNA extraction from FFPE tissue was performed using RNeasy® FFPE Kit (Qiagen Germany) in which the total RNA fraction contains RNA fragments down to 18 nucleotides in length. RNA260/280 ratio and the RNA concentration of each sample was measured using NanoDrop (Tecan).

### miRNA expression profiling

miRNA expression profiling was carried out using Taq-man Low Density Array Human microRNA A + B Cards v3.0 (Micro fluid cards, Applied Biosystems, Foster City, USA). Equal volumes of flow-through were used as input material, and the miRNA was reverse transcribed using Megaplex^tm^ pool A and B (Applied Biosystems) followed by pre amplification. The cDNA pool was applied to the multiplex array cards and the experiment was run on the ABI 7900 HT platform using cycling conditions as provided by the manufacturer. Normalization of results was done using RQ Manager (Applied Biosystems). A common threshold was established across all array cards for each miRNA, expression levels were normalized to MammU6 and average delta-Ct was used in downstream analyses. miRNA expression data is available at GEO (NCBI) with series accession no. GSE62610.

A total of 350 ng total RNA was reverse transcribed using Megaplex^tm^ pool A (Applied Biosystems) followed by pre-amplification according to manufacturer protocol for miRNA expression profiling in FFPE tissue. We performed singleplex RT-PCR amplifications for each of the candidate miRNAs selected for validation using Taq-man probes with the assay ids: 001129, 002255, 002306, 002295, 001608 and 001960, with MammU6 as reference. All samples were measured in triplicates and a no template and a no RT control were included on all plates.

### Statistical analyses

Statistical analyses were performed using STATA version 10.1 (StataCorp, College Station TX, USA). P-values < 0.05 were considered statistically significant. Ranking of the miRNAs was done using Mann–Whitney rank-sum test in the screening study. Median fold change was given by the relation between the median expression level in the recurrent group versus the level in the non-recurrent group. Association with biochemical recurrence after radical prostatectomy was analyzed using univariate and multivariate Cox regression analysis in the validation study. For each variable in the Cox regression analyses the proportional hazard assumption was verified by log-log survival curves. The prediction accuracy was estimated using Harrell c’s concordance index. Pathological T-stage was dichotomized in localized and extra prostatic disease. Gleason score was grouped in three categories containing scores 5–6, 7, and 8–10 respectively. Preoperative PSA levels were grouped according to the D’Amico classification with <10 ng/ml, 10 to 20 ng/ml and >20 ng/ml.

## Results

In total, 36 tumor samples were laser micro dissected and 672 miRNAs profiled using multiplex RT-qPCR to identify novel miRNAs associated with recurrence after RP. Sixty % of the patients had suffered recurrence and the median follow-up of those without recurrence was 66 months (range 31–80 months). Clinical and histopathological information is listed in Table 1. Initially the miRNA expression data was filtered to exclude miRNAs with no detection above background in any of the samples, leaving 536 miRNAs for further analysis. A total of 235 (44%) miRNAs were detected in all samples in the filtered dataset.

**Table 1:** **Clinical and histopathological characteristics of the screening cohort and the validation cohort**

Clinical variable	Screening cohort (FF)	Validation cohort (FFPE)
Total number of cancer samples	36	163
Age median(range) Years	63 (46–71)	62(48–72)
Gleason grade		
Low (5–6)	17 (47%)	60 (37%)
Intermediate (7)	15 (42%)	85 (52%)
High (8–10)	4 (11%)	18 (11%)
Pathological stage		
T2a-c	19 (53%)	96 (59%)
T3a-b	17 (47%)	67 (41%)
Time to recurrence (range) Months	15.6 (1–74)	24 (3–122)
Follow up non-recurrent cases Months	66 (31–80)	65 (48–114)
Recurrence		
Yes	22 (61%)	96 (59%)
No	14 (39%)	67 (41%)
Margin status		
Positive	16 (44%)	45 (28%)
Negative	20 (56%)	118 (72%)
Pre-operative PSA (range)	16.0 (5.3-42.5)	13.2 (2.1-64.5)

FF: fresh frozen tumors. FFPE: formalin fixed paraffin embedded tumors.

### Delineation of key miRNA transcripts associated with outcome

We delineated miRNAs that showed significant association with recurrence. In total, 28 miRNAs were found to be significantly up regulated and 3 miRNAs were significantly down regulated in tumors from patients with recurrence (p < 0.05 Mann–Whitney), compared to tumors from patients without recurrence. Median fold changes between recurrent and non-recurrent cases ranged from 1.18 to 16 in the up regulated miRNAs and 4.2 for the down regulated miR-24-1-5p (Table 2). Top ranked up regulated miRNA was miR-449b (p = 0.0061, Mann–Whitney) with a 2.8 times higher expression in patients with recurrent disease compared with patients with non-recurrent disease.

**Table 2:** **miRNAs significantly associated with recurrence**

miRNA	Rank sum p-value	Reference	Median fold change	Selected for validation
Up regulated in recurrent cases				
mir449b	0.0061	[16, 22]	2.80	X
mir137	0.0069		14.88	X
mir30e-3p	0.0195		1.59	
mir339-3p	0.0195		1.77	
mir362-5p	0.0195		2.38	
mir630	0.0202		1.00	
mir149	0.0212		1.98	X
mir342-3p	0.0231		1.75	
mir30a-3p	0.0231		1.63	
mir301b	0.0252		1.98	
mir182	0.0252		1.83	
mir484	0.0252	[16]	1.71	
mir126	0.0252	[20]	2.34	
mir223	0.0297		2.01	X
mir636	0.0297		2.23	
mir615-3p	0.0315		Na.	X
mir622	0.0335		5.69	
mir548c-3p	0.0339		Na.	
mir197	0.0378		1.70	
mir616-5p	0.042		2.16	
mir367	0.0426		3.67	
mir214	0.0442		2.10	X
mir125a-5p	0.0442	[20]	1.34	
mir32	0.0442		1.56	
mir566	0.0449		Na.	
mir500*	0.0461		5.22	
mir10a	0.0478	[20]	2.06	
mir10b	0.0478	[20, 22]	1.94	
Down regulated in recurrent cases				
mir24-1*	0.0298		3.43	
mir154	0.0353	[19]	na.	
mir873	0.0406		na.	

### Validation in independent cohort

No miRNAs were significantly associated with recurrence when using conservative Bonferroni correction for multiple testing corrections – likely due to the small initial patient cohort. To compensate for this we performed an independent validation of the most significant miRNAs. Initially we selected a subset of 40 patients (20 recurrent and 20 non-recurrent patients) to investigate candidate miRNAs before testing the miRNAs using the whole cohort. Criteria for selecting miRNAs for validation were the p-value in rank-sum test, fold change, biological function determined in the literature, and finally for technical reasons that the assay was included in the Megaplex pool A used for cDNA synthesis and pre-amplification. Based on these criteria, six miRNAs were selected for validation: miR-449b, miR-137, miR-149, miR-214, miR-223 and miR-615-3p. We found miR-449b expression to be associated with recurrence status in the 40 patients (p = 0.017, Chi^2^-test). MiR-137 and miR-615-3p failed qPCR amplification, and there were no significant association between expression level of the miRNA and recurrence status for the rest (data not shown). Consequently, only miR-449b was measured in the entire validation cohort.

**Figure 1:**
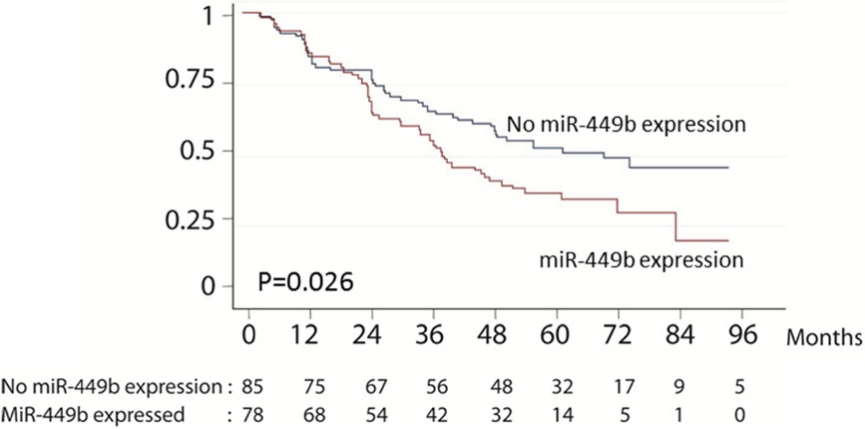
**Kaplan-Meier survival curves showing recurrence free survival as function of miR-449b expression in the validation cohort.**

The entire validation cohort consisted of RNA samples extracted from FFPE tissue samples originating from 163 patients who underwent radical prostatectomy. Clinical characteristics of the validation cohort are summarized in Table 1. Of the 163 samples, 78 (48%) of the samples had detectable miR-449b expression above background. We found a significant association between miR-449b expression and PSA group (p = 0.02; chi2), but no association with other clinical variables as shown in Table 3. Using univariate Cox regression analysis, we found that expression of miR-449b was significantly associated with the risk of recurrence after RP (HR = 1.57, p = 0.027). This correlation was also observed from Kaplan-Meyer survival estimates (Figure 1). In multivariate Cox regression analysis modeled with Gleason grade, pathological t-stage, margin status, age and preoperative PSA, we found that miR-449b expression was an independent predictor of recurrence after RP (HR = 1.90, p = 0.003) (Table 4). The overall prediction accuracy determined by the Harrell´s C index of the multivariate model containing clinical variables alone was 0.69, compared to 0.71 when miR-449b expression status was added to the model.

**Table 3:** **miR-449b expression in relation to the clinical variables in the validation cohort**

Clinical variable	Chi ^2^–test	miR-449b expression	No miR-449b expression
	P-value		
Total number of cancer samples		78	85
Gleason grade	0.27		
Low (5–6)		27	33
Intermediate (7)		45	40
High (8–10)		6	12
Pathological stage	0.73		
T2a-c		47	49
T3a-b		31	36
Pre-operative PSA	0.02		
0-10 ng/ml		22	27
10-20 ng/ml		44	31
20- ng/ml		12	27
Recurrence	0.05		
Yes		26	41
No		52	44
Margin status	0.21		
Negative		54	64
Positive		24	19

**Table 4:** **Univariate- and multivariate analysis of recurrence free survival in the validation cohort**

	Univariate			Multivariate		
	HR (95% confidence interval)	p-value	PA %	HR (95% confidence interval)	p-value	PA %
miR-449b expression	1.57 (1.05-2.37)	**0.028**	0.55	1.90 (1.25-2.85)	**0.003**	
Organ confined	2.76 (1.83-4.16)	**<0.001**	0.61	2.57 (1.66-3.07)	**<0.001**	
Gleason grade	1.64 (1.21-2.23)	**0.002**	0.58	1.53 (1.08-2.15)	**0.017**	
PSA	1.88 (1.41-2.50)	**<0.001**	0.63	1.74 (1.30-2.33)	**<0.001**	
Margin status	1.14 (1.02-1.28)	**0.027**	0.62	1.06 (0.91-1.23)	0.7	
Age	1.01 (0.97-1.05)	0.557	0.51	1.02 (0.98- 1.06)	0.365	
Clinical model						0.69
Clinical model with miR-449b expression						0.71

Values in bold indicate significant uni- and multivariate analysis (P<0.05).

## Discussion

Our study revealed 31 miRNAs differentially expressed between patients suffering recurrence and patients with no recurrence. Furthermore the top ranked miR-449b was successfully validated in an independent cohort of 163 patients using RT-qPCR and found to be an independent predictor of biochemical recurrence after prostatectomy.

miRNA in relation to cancer constitutes an interesting field of research due to their role in gene expression regulation and because they are generally more stable than mRNA. There is however a number of conflicting results from miRNA profiling studies including irreproducible results. One major issue is the sample itself, and the tissue actually being profiled. Samples where the carcinoma cell percentage is low, will inevitably reveal a miRNA profile which is a mix the miRNAs from the carcinoma cells and the profile from the surrounding normal tissue, thus diluting the miRNA expression in the carcinoma cells. Regarding studies of aggressive PC, tumor heterogeneity has often been overlooked. Normally only a single tumor biopsy is used and the miRNA profile produced is a measurement of the miRNA expression in that given part of the tumor. If the tumor contains more aggressive clones in other parts of the prostate a discrepancy will arise between the clinical performance of the tumor and the miRNA profile obtained from the more indolent tumor cells. Another issue influencing reproducibility is the technical differences between studies. Although good reproducibility exist within RT-qPCR profiling platforms, the correlation with microarray platforms is often not high, as reported by Chen et al. [26]. The number of miRNAs actually being profiled also differs between studies ranging from 119 [19] to 676 [22], in addition since different platforms are used the specificity and sensitivity of the probes detecting the miRNAs can differ leading to greater variability of the expression measurements. However, in spite of these challenges, several previously identified miRNAs associated with adverse characteristics in PC were also identified in our study. The miRNAs, miR-449b and miR-10b were seen previously by Fendler et al. [22], differentiating between early or late recurrence. Furthermore, several miRNAs (miR-126, miR-125-5p, miR-10a/b) overlapped with the miRNAs identified by Prueitt et al. [20] being associated with perineural invasion. Finally, miR-449b and miR-484 overlapped with the miRNAs associated with extraprostatic disease extension identified by Ambs et al. [16]. Among the down regulated microRNAs found in this study, miR-154 was also seen down regulated in patients with biochemical recurrence after prostatectomy by Tong et al. [19]. In addition to the microRNAs previously linked to aggressive traits in PC several new microRNAs were identified to be associated with recurrent disease.

The use of biochemical recurrence as surrogate end-point of aggressive prostate cancer has limitations. Other end-points like time to metastasis or progression to death are stronger end-points for the clinical aggressiveness of prostate cancer, unfortunately this data was not available for the cohort. The Gleason grade included in the multivariate model of the validation cohort is based on the original pathology grading. Gleason grading has changed over time and a re-evaluation of the prostatectomy specimens would strengthen the model.

The miR-449b gene is situated on chromosome 5 in the second intron of the CDC20B gene. MiR-449b shares seed sequence with miR-449a which is also transcribed in the second intron of CDC20B. This off course implies similar mRNA targets and thus similar functions. In our study we found that miR-449a had a similar fold change difference of 3.8 compared with 3.2 for miR-449b between the recurrent and non-recurrent cases, but miR-449a expression was not significantly associated with recurrence in Mann–Whitney test. This difference can be due to specificity and/or sensitivity of the PCR probes for miR-449a and miR-449b respectively.

As described above the miR-449 cluster has previously been associated with adverse outcomes in PC. In contrast to this miR-449 functional studies have provided conflicting results. The miR-449 cluster has been implicated in different cellular functions primarily in cell cycle control and in cellular differentiation [27]. MiR-449b has been shown to inhibit androgen receptor expression resulting in inhibited androgen mediated proliferation [28]. Studies using the Saos-2 osteosarcoma cell line show that mir-449 cluster transcription is inducible by the E2F1 transcription factor. Induced miR-449 has been shown to cause causes cell cycle arrest and promote apoptosis by inhibiting CDK6 and CDC25A. In this way it functions as a negative feedback mechanism against E2F1 induced cell proliferation [28]. In other studies using cell lines originating from prostate, breast and lung cancer the miR-449 cluster has also been associated with cell cycle arrest and a role as tumor suppressor although through different mechanisms [29–31]. However a different functional role of miR-449 cluster is the interaction with LEF-1 a known effector of the WNT pathway [32]. In the cell line hBM-MSC the miR-449 cluster has been shown to directly inhibit the LEF-1 gene and thus the WNT pathway. Although WNT pathway inhibition in general inhibits cancer growth, including PC, [33–35] other effects have been observed that relate to PC progression. Firstly LEF-1 inhibition has been shown to inhibit osteoblast differentiation and result in reduced bone density, a phenotype that would facilitate the establishment of PC cells in bone [36]. Secondly studies looking at the interaction between WNT signaling and androgen receptor function reveals a complex interaction with some evidence that WNT pathway signaling enhances androgen receptor signaling other evidence inhibiting androgen signaling [37]. The miR-449 cluster has also been identified targeting the NOTCH pathway thus altering cell differentiation [38]. Whereas NOTCH pathway signaling again in general is shown to induce EMT and cancer progression, NOTCH signaling is also linked to inhibiting bone formation. In this manner and like the inhibition of LEF-1, the mechanism of action for miR-449b overexpression in aggressive PC could be an increased propensity to metastasize to bone. Further studies are needed to investigate the functional role of miR-449 in normal and malignant prostate cells.

## Conclusion

The current study identified 31 miRNAs to be associated with biochemical recurrence of prostate cancer after radical prostatectomy. The highest ranked miRNA, miR-449b, was further validated in an independent cohort of patients and shown to be an independent predictor of recurrence after radical prostatectomy.
